# A Preliminary Study of Muscular Artifact Cancellation in Single-Channel EEG

**DOI:** 10.3390/s141018370

**Published:** 2014-10-01

**Authors:** Xun Chen, Aiping Liu, Hu Peng, Rabab K. Ward

**Affiliations:** 1 Department of Biomedical Engineering, School of Medical Engineering, Hefei University of Technology, Hefei 230009, China; E-Mail: xunchen@ece.ubc.ca; 2 Department of Electrical and Computer Engineering, University of British Columbia, Vancouver, BC V6T 1Z4, Canada; E-Mails: aipingl@ece.ubc.ca (A.L.); rababw@ece.ubc.ca (R.K.W.)

**Keywords:** EEG, single-channel, muscular artifacts, EEMD, JBSS

## Abstract

Electroencephalogram (EEG) recordings are often contaminated with muscular artifacts that strongly obscure the EEG signals and complicates their analysis. For the conventional case, where the EEG recordings are obtained simultaneously over many EEG channels, there exists a considerable range of methods for removing muscular artifacts. In recent years, there has been an increasing trend to use EEG information in ambulatory healthcare and related physiological signal monitoring systems. For practical reasons, a single EEG channel system must be used in these situations. Unfortunately, there exist few studies for muscular artifact cancellation in single-channel EEG recordings. To address this issue, in this preliminary study, we propose a simple, yet effective, method to achieve the muscular artifact cancellation for the single-channel EEG case. This method is a combination of the ensemble empirical mode decomposition (EEMD) and the joint blind source separation (JBSS) techniques. We also conduct a study that compares and investigates all possible single-channel solutions and demonstrate the performance of these methods using numerical simulations and real-life applications. The proposed method is shown to significantly outperform all other methods. It can successfully remove muscular artifacts without altering the underlying EEG activity. It is thus a promising tool for use in ambulatory healthcare systems.

## 1. Introduction

The electroencephalogram (EEG) signals are often contaminated by various physiological activities of non-interest, such as the electrocardiogram (ECG), electrooculogram (EOG) and electromyogram (EMG). While ECG and EOG artifacts can be effectively removed by using adaptive filters and blind source separation (BSS) techniques [1], the artifacts induced by muscular activity (e.g., biting, chewing and frowning) are particularly difficult to correct [2]. The main reason lies in the fact that EMG artifacts have a higher amplitude than the EEG signals, a wide spectral distribution and a variable topographical distribution [2]. These muscular artifacts obscure the EEG signals and make EEG interpretation extremely complicated or almost impossible [3].

Low-pass filters are commonly employed to remove muscular artifacts. However, since the frequency spectrum of muscular artifacts significantly overlaps with that of brain signals, these filters unfortunately suppress the brain signals of interest during the suppression of the muscular artifacts [4]. Recently, using the popular BSS technique, independent component analysis (ICA) has been extensively explored for this purpose [5–7]. ICA utilizes higher-order statistics to separate the EEG recordings into statistically independent components (ICs). Clean EEG data can then be reconstructed by removing the artifact-related ICs from the raw EEG data. In some studies, however, muscular artifacts seriously contaminate most ICs. This results in a clearly observable crosstalk between brain and muscle activities [8,9]. One possible reason is that ICA only exploits the spatial structure of source signals. Thus, it is suitable when source signals are temporally statistically independent [10]. However, artifacts typically have certain temporal structures, which can be exploited for better source separation. To this end, a canonical correlation analysis (CCA) method has been proposed as a more suitable BSS approach for separating EMG artifacts from EEG signals [11]. Due to their broad frequency spectrum, EMG artifacts resemble temporal white noise and, thus, have lower autocorrelation compared to EEG signals. The CCA method exploits this characteristic for EMG cancellation and has been shown to outperform ICA on simulated data. Later, these results were also been documented by Gao *et al.* [12].

In recent years, biomedical signal measurement and processing techniques have been increasingly deployed in ambulatory situations, particularly in healthcare applications, where minimal instrumentation and low computational complexity are required [13–15]. To reduce the complexity, many ambulatory systems use only one single EEG channel [14,15]. However, almost all current methods for muscular artifact cancellation have been designed to handle multichannel EEG datasets and will fail to isolate the muscle activity in situations where only single-channel EEG recordings are available.

To address this issue, we propose a simple, yet effective, method to achieve muscular artifact cancellation in single-channel EEG cases. This method has a two-step strategy. The first step decomposes the single-channel EEG into multichannel datasets. To implement this step, empirical mode decomposition (EMD) is a suitable option. EMD is a single-channel technique that decomposes nonstationary and nonlinear time series into a finite number of intrinsic mode functions (IMFs) [16]. Compared with other decomposition methods (e.g., wavelet transform), EMD is completely data-driven, *i.e.*, it decomposes a signal without requiring prior knowledge. It has been shown to be efficient in many biomedical applications, e.g., denoising electrohysterogram (EHG) signals [17] and removing eye blink artifacts from EEG recordings [18]. It should be noted that a noise-assisted version of EMD, called ensemble EMD (EEMD), was recently proposed and shown to have better performance than the original EMD [19]. EEMD extracts IMFs in a manner such that the mode mixing disadvantage of the EMD method is corrected. Sweeney *et al.* has utilized this new decomposition method with CCA to remove the motion artifacts from functional near-infrared spectroscopy (fNIRS) and EEG data [20].

In the second step, the emerging joint BSS (JBSS) techniques are formulated to separate the muscle artifacts from the multidimensional datasets obtained in the first step. JBSS algorithms attempt to achieve blind source separation on multiple datasets simultaneously by balancing two criteria: (1) maximizing the independence of the estimated sources within each dataset; and (2) maximizing the source dependence across datasets. To utilize JBSS for the blind source separation purpose, the original dataset and its time-delayed version are used as the input to the JBSS methods. The advantage of using the JBSS techniques instead of the BSS methods is that besides extracting statistically independent or uncorrelated sources, JBSS also exploits the temporal structure of the sources by examining their dependence with their time-delayed version. When explored by second order statistics (SOS), the stronger dependence indicates higher autocorrelation. Thus, the separation of muscle and brain activity components can be achieved due to the relative low autocorrelation of muscular artifacts in comparison with brain activity [11]. In this work, the two most popular JBSS methods, CCA and independent vector analysis (IVA) [21,22], will be explored with EEMD. While both CCA and IVA exploit SOS for the dependence, CCA and IVA separately employ SOS and higher order statistics (HOS) for source estimation. We denote the two EEMD-JBSS combinations as EEMD-CCA and EEMD-IVA, respectively.

In this paper, we also conduct a comparison study by examining other possible single-channel techniques, which have been devised for other purposes. Single-channel ICA (SCICA) is an adaptation of ICA to single-channel signals [23]. This method assumes that the signal is stationary and is composed of spectrally disjoint sources. The combination of EEMD and ICA, denoted as EEMD-ICA, is another popular method developed for source separation of single-channel recordings [24]. The main contribution of this work are the proposed practical solutions for the muscular artifact cancellation problem in single-channel EEG. This is of special importance at present, as ambulatory healthcare continues to draw increasing attention.

We examine the performance of the proposed EEMD-JBSS methods on both synthetic data and real data. We first validate the methods on simulated data. We then apply them to a real ictal EEG dataset and a real EEG dataset collected from subjects, while riding a stationary bicycle. The EEG signals are contaminated with muscular artifacts. We note that while the EEMD-JBSS method has been proposed to remove muscle activity from the single-channel EEG case, it is generally applicable to cases when one dataset contains relatively few channels (e.g., two or three). This is done by first applying EEMD to each channel and then utilizing JBSS on the integrated signals after decomposition.

## 2. Materials and Methods

### 2.1. Methods

In this section, we first briefly introduce the existing techniques. Then, we describe the proposed two EEMD-JBSS methods.

Notations: Scalars are denoted by lowercase italic letters (*a, b, etc*.), vectors by lowercase boldface letters (**a**, **b**, *etc.*), matrices by boldface capitals (**A**, **B**, *etc.*) and the number of rows and columns by italic capitals (*T, N, etc.*). A matrix or vector transposition is denoted by an uppercase superscript *T* (e.g., **X***^T^*, **v***^T^*). The vector **x** (with size 1 × *T*) is used to represent the original single-channel signal with **x**(*t*) (*t* = 1, 2,…, *T*) denoting the signal value at the time point *t*.

#### 2.1.1. Independent Component Analysis

As ICA is a well-known BSS technique in the literature, we only briefly describe its basic concept. Suppose the mixed signals are stored in one matrix, **X**, with size *P* × *T*, where *P* indicates the number of channels and *T* indicates the number of observations per channel. The goal of ICA is to separate the mixed signals **X** into their independent sources **S** without any other prior knowledge using the linear model:

(1) X=AS

where **A** is the mixing matrix and **S** is of dimension *P* x *T*. It is possible to estimate the underlying sources from the mixture signals provided they are statistically independent. Several algorithms have been developed to solve this problem. In this study, we employed the popular FastICA algorithm [25]. It is based on a fixed-point iteration scheme for maximizing the non-Gaussianity of the sources. Using this algorithm, the mixing matrix **A** and the underlying sources **Ŝ** can be estimated. Those sources that are deemed to be artifacts can be removed by setting the corresponding row of the matrix **Ŝ** to zero. The artifact-free signals can then be reconstructed.

#### 2.1.2. Single-Channel ICA

SCICA is an adaptation of ICA to single-channel signals [23]. The algorithm is described as follows. First, the observed signal **x** is broken up into a sequence of contiguous blocks **b**(*k*) of length *N*:

(2) $$\text{b}(k)={\left[\text{x}(k\tau ),\cdots ,\text{x}(k\tau +N-1)\right]}^{T}$$

where *k* is the block index, *τ* is a time delay and (*Kτ* + *N* − 1) is the length of the original signal. Then, the matrix **X** is formed as a set of observations *b*(*k*) (*k* = 1, 2,…, *K*) as below:

(3) $$\text{X}={\left[\text{b}(1),\ldots ,\text{b}(K)\right]}^{T}$$

It should be noted that the performance of SCICA significantly depends on the parameters chosen. The authors in [23] suggest that the users select those parameters empirically. Finally, the FastICA algorithm can be applied to the matrix **X** to obtain the mixing matrix **A** and the underlying sources **Ŝ**. From the above procedure, it can be seen that SCICA assumes the signal **x** to be stationary and composed of spectrally disjoint sources. These assumptions, however, do not always hold in practical applications.

#### 2.1.3. Canonical Correlation Analysis

Suppose two zero-mean datasets are stored in two matrices, **X**_1_ with size *P*_1_ × *T* and **X**_2_ with size *P*_2_ × *T*, where *P*_1_ and *P*_2_ indicate the numbers of channels in **X**_1_ and **X**_2_, respectively, and *T* denotes the number of observations per channel. The aim of CCA is to find linear combinations of both **X**_1_ and **X**_2_ channels that have the maximum correlation coefficient with each other [26]. This leads to the following objective function with constraints:

(4) $$\begin{array}{cc} \underset{{\text{v}}_{1},{\text{v}}_{2}}{\max} & {\left({\text{v}}_{1}^{T}{\text{X}}_{1}{\text{X}}_{2}^{T}{\text{v}}_{2}\right)}^{2}\\ \text{s.t}. & \begin{array}{cc} {\text{v}}_{1}^{T}{\text{X}}_{1}{\text{X}}_{1}^{T}{\text{v}}_{1}=1, & {\text{v}}_{2}^{T}{\text{X}}_{2}{\text{X}}_{2}^{T}{\text{v}}_{2} \end{array}=1 \end{array}$$

where **v***_i_*’s (*i* = 1, 2) are the weight vectors.

The solutions to this problem are the eigenvectors of the matrices ${\left({\text{X}}_{1}{\text{X}}_{1}^{T}\right)}^{-1}{\text{X}}_{1}{\text{X}}_{2}^{T}{\left({\text{X}}_{2}{\text{X}}_{2}^{T}\right)}^{-1}{\text{X}}_{2}{\text{X}}_{1}^{T}$ and ${\left({\text{X}}_{2}{\text{X}}_{2}^{T}\right)}^{-1}{\text{X}}_{2}{\text{X}}_{1}^{T}{\left({\text{X}}_{1}{\text{X}}_{1}^{T}\right)}^{-1}{\text{X}}_{1}{\text{X}}_{2}^{T}$, respectively. The canonical variates (CV) **U***_i_*’s (*i* = 1, 2) can be calculated directly from the original matrices **X***_i_*’s as ${\text{U}}_{i}={\text{V}}_{i}^{T}{\text{X}}_{i}$. The corresponding rows of **U**_1_ and **U**_2_ are highly correlated, while the rows within each individual **U***_i_* are uncorrelated with each other. The detailed derivation can be referred to [27].

CCA has been further extended to solve the BSS problem in a functional magnetic resonance imaging (fMRI) study by assuming the source components to be maximally autocorrelated and mutually uncorrelated [28]. In this setting, let **X**_1_ be the observed data matrix **X** with *P* mixtures and *T* samples, and let **X**_2_ be a temporally delayed version of the original data matrix **X**_2_(*t*) = **X**(*t* − 1). Thus, CCA can separate the recorded data into the self-correlated and mutually uncorrelated sources. As a potential alternative for the most widely used ICA method, CCA has been previously tested with a number of ICA algorithms. The CCA-based methods were shown to outperform the ICA-based techniques for EEG/fNIRS artifact removal [11,12,20]. Due to the usage of second-order statistics (SOS), they were more computationally efficient when having similar qualitative results for EEG/fMRI source separation [28,29].

#### 2.1.4. Independent Vector Analysis

IVA is an extension of ICA from one to multiple datasets. In [21], IVA was formulated as a general JBSS framework to ensure that the extracted sources are independent within each dataset and well correlated across multiple datasets. In IVA, the concept of source component vector (SCV) is defined across multiple datasets [30]. The *p*-th SCV, ${\text{s}}_{p}={\left[s_{p}^{\left[1\right]},s_{p}^{\left[2\right]},\ldots ,s_{p}^{\left[M\right]}\right]}^{T}(p=1,2,\ldots ,P)$, is arandom vector independent of all other SCVs and the components within each SCV are dependent. The symbol $s_{p}^{\left[m\right]}$ represents the *p*-th underlying source component in the *m*-th dataset. The goal of IVA is to identify the independent SCVs from multiple multidimensional datasets. This can be achieved by minimizing the mutual information among the estimated SCVs **ŝ***_p_*’s [21]:

(5) $$\begin{array}{cc} I_{IV\;A} & ≜I[{\hat{\text{s}}}_{1};{\hat{\text{s}}}_{2};\ldots ;{\hat{\text{s}}}_{P}]\\ & =\overset{P}{\underset{p=1}{\text{∑}}}H[{\hat{\text{s}}}_{p}]-H[{\hat{\text{s}}}_{1},{\hat{\text{s}}}_{2},\ldots ,{\hat{\text{s}}}_{P}] \end{array}$$

where *H* denotes the entropy. The detailed derivation can be found in [21]. By solving the above optimization problem, each estimated SCV **ŝ***_p_* is independent of all other estimated SCVs, and meanwhile, the components within each SCV are dependent, e.g., $s_{p}^{\left[1\right]}$ and $s_{p}^{\left[2\right]}$ are highly correlated.

The implementation algorithms involve the selection of specific probability distributions for the SCVs. The most popular methods include IVA-L [30] and IVA-G [21]. IVA-L assumes that each SCV follows a multivariate Laplace distribution that is isotropic and possesses no second-order correlation, while IVA-G exploits second-order statistical information across datasets by assuming that each SCV is multivariate Gaussian distributed. In some applications, the second-order information across datasets may be minimal, such as in the frequency domain BSS for speech recognition. However, in some other applications, it is expected to have a much larger correlation, for instance in group fMRI studies. In this work, we will utilize IVA-G by taking into account the importance of the second-order information, which can exploit the temporal structure of muscular artifacts. Therefore, with a similar setting in CCA, IVA can separate the recorded data into the self-correlated and mutually-independent sources. The possible advantage of IVA over CCA is that IVA is able to extract independent sources rather than uncorrelated ones by using HOS. However, this requires more computational time. Moreover, IVA assumes the underlying sources to follow specific distributions, which may not be true in practice.

#### 2.1.5. Ensemble Empirical Mode Decomposition

EMD is a single-channel decomposition method for nonstationary and nonlinear signals [16]. EMD decomposes a signal into a finite number of IMFs that represent fast to slow oscillations. An IMF is a function that satisfies two conditions [16]: (1) the number of extrema and the number of zero crossings must either be equal or differ by at most one; and (2) at any point, the mean value of the envelope defined by the local maxima and the envelope defined by the local minima is zero. To obtain an IMF from the original signal **x**, a sifting process is performed [16]. First, all extrema of the original signal **x** need to be identified. All local maximum points are connected by a cubic spline line to form the upper envelope **e***_u_*. Additionally, all local minimum points are connected similarly to form the lower envelope **e***_l_*. The mean of **e***_u_* and **e***_l_*, **a**_1_, is calculated as:

(6) $${\text{a}}_{1}=\frac{{\text{e}}_{u}+{\text{e}}_{l}}{2}$$

The difference between the signal and the mean is defined as the first component **h**_1_ as:

(7) $${\text{h}}_{1}=\text{x}-{\text{a}}_{1}$$

In the second sifting process, **h**_1_ is treated as the signal, and the mean **a**_11_ of its local maxima and local minima is found. We then have:

(8) $${\text{h}}_{11}={\text{h}}_{1}-{\text{a}}_{11}$$

Subsequently, we can repeat this sifting procedure *k* times until **h**_1_*_k_* is an IMF, with:

(9) $${\text{h}}_{1k}={\text{h}}_{1(k-1)}-{\text{a}}_{1k}$$

Therefore, the first IMF component derived from the original signal is designated as:

(10) $${\text{c}}_{1}={\text{h}}_{1k}$$

A criterion for stopping the sifting process when obtaining an IMF has been established by limiting the size of the standard deviation (SD), calculated from the two consecutive sifting sequences as below:

(11) $$\text{SD}=\overset{T}{\underset{t=1}{\text{∑}}}\left\{\frac{{\left[{\text{h}}_{1(k-1)}(t)-{\text{h}}_{1k}(t)\right]}^{2}}{{\text{h}}_{1(k-1)}^{2}(t)}\right\}$$

A typical value for SD can be set between 0.2 and 0.3 [16].

To extract the 2nd IMF component, we remove *c*_1_ from the original signal *x*:

(12) $${\text{r}}_{1}=\text{x}-{\text{c}}_{1}$$

The residual *r*_1_ is treated as a new signal, and the same sifting process is applied to obtain the 2nd IMF component *c*_2_ and the residual:

(13) $${\text{r}}_{2}={\text{r}}_{1}-{\text{c}}_{2}$$

This procedure is repeated on the subsequent residuals **r***_j_*’s, until the final residual **r***_J_* no longer contains any oscillation information,

(14) $${\text{r}}_{j}={\text{r}}_{j-1}-{\text{c}}_{j}$$

By summing up Equations (12)–(14), we can obtain:

(15) $$\text{x}=\overset{J}{\underset{j=1}{\text{∑}}}{\text{c}}_{j}+{\text{r}}_{J}$$

Thus, we decompose the original signal **x** into *J* empirical modes **c***_j_*’s and a residue **r***_J_*.

However, the original EMD algorithm is highly sensitive to noise. Recently, Huang *et al.* introduced a new noise-assisted data analysis method, called EEMD [19]. The method defines the true IMF components as the mean of an ensemble of trials. Each trial consists of the signal plus an additive independent identically distributed white noise of the same standard deviation. In this case, although each individual trial may produce noisy results, the noise is canceled out in the ensemble mean of sufficient trials, since the noise in each trial is assumed independent. Regarding the ensemble number *I*, it is found that the performance of the technique becomes fairly consistent when using ten or more ensembles in our application. This is a acceptable number in practice considering the computational cost. The noise standard deviation has been suggested empirically to be 0.2-times the standard deviation of the original signal [19].

#### 2.1.6. EEMD-ICA

The idea of combining EEMD with ICA for source separation from single-channel recordings was first proposed in [24] and was employed for the removal of ECG from EMG and also EMG/EOG artifacts from EEG. This is the only work we have found related to muscular artifact cancellation in single-channel EEG. However, the relevant results were limited. In this method, the EEMD technique can be used to create a multichannel signal matrix **X**, comprised of IMFs from a single-channel recording **x**. This matrix **X** can then be employed as the input to the FastICA algorithm with the aim of estimating the underlying sources **ŝ**. The sources deemed as artifacts can be removed by setting the corresponding row of the matrix **ŝ** to be zero. The source matrix is then passed through the mixing matrix **A** to return the cleaned multichannel signals **X̂**, which are now, ideally, free of artifacts. The artifact-free single-channel recording **x̂** can be determined by summing the recovered IMFs in the matrix **X̂**.

#### 2.1.7. The Proposed EEMD-JBSS

To deal with the muscular artifact cancellation problem in single-channel EEG, we propose taking advantage of both EEMD and JBSS by exploring their combination. In fact, we propose a two-step strategy, operating in a similar manner to the EEMD-ICA technique. In the first step, EEMD is employed to decompose the single-channel EEG signal **x** and to derive a set of averaged IMFs. All of the IMF components and the final residual are placed into a matrix **X**. The size of **X** is *P × T*, where *P* = *J* + 1. In the second step, the matrix **X** and its temporally delayed version matrix **X**(*t* − 1) are employed as the input to CCA or IVA. Then, the underlying sources **Ŝ** in **X** can be extracted and ordered in terms of their autocorrelations from high to low. The sources with low autocorrelation correspond to muscular artifacts and can be removed by setting the corresponding row of the matrix **Ŝ** to be zero. The artifact-free multichannel signals **X̂** can be reconstructed by using the updated source matrix and the mixing matrix **A**. The recovered single-channel signal without muscular artifacts **x̂** can be determined by simply summing the new IMFs components in the matrix **X̂**. After these two steps, the muscle activity is removed from the single-channel EEG.

### 2.2. Data Description

#### 2.2.1. Synthetic Data

To demonstrate the performance of the proposed EEMD-JBSS methods, we generated synthetic single-channel EEG signals with two types of muscular artifacts. We employed some measures to test the performance, since the ground truth is known.

Traditionally, the “ground truth” EEG signals without muscular artifacts are selected by visual inspection of experienced neurophysiologists. However, not only is it difficult to obtain clean EEG signals, but there is also no guarantee that the signals are completely free of muscle activity when relying solely on visual inspection. Thus, in this study, we tend to use synthetic EEG data. A single-channel EEG data series can be generated according to the phase-resetting theory [31,32]. Similar to Makinen *et al.* [31], we generated our simulated data by summing 4 such sinusoids, whose frequencies were chosen randomly in the range 4-20 Hz. The sampling frequency was 250 Hz. Ten trial EEG data were generated, and each trial dataset was 1 s long. Thus, a 10-s series **x***_EEG_* could be formed by concatenating the 10 trial datasets, containing mainly theta, alpha and beta activities. It should be noted that while each trial dataset included 4 distinct frequencies, the frequencies chosen for different trial data were also independent, which means that there was rich frequency information in the 10-s series.

To simulate real-life situations, obtaining pure muscle activity is necessary. It is insufficient to distinguish muscular artifacts directly from the EEG signal, as it contains both muscle and brain activity. To remove the brain activity and acquire the muscle activity, ICA was utilized to decompose a real EEG dataset with 21 channels. A neurophysiologist labeled the eye blink artifacts, eye movement artifacts and muscular artifacts from all of the decomposed ICs by inspecting some features, such as the power spectral density and topography. It is important to note that a large number of ICs contained both EMG and ongoing EEG activities. Nevertheless, there existed one component containing pure EMG activity, denoted by **x***_EMG_*. Since we focus on single-channel issues, it is not necessary to reconstruct the component with the corresponding field distribution.

**Simulated muscle activity:** To extensively investigate the performance of the methods, a mount of synthetic muscle artifacts have also been generated according to the work of Delorme *et al.* [33]. The muscle activity was modeled using random noise, band-pass filtered between 20 and 60 Hz. In this study, we generated 100 independent transient muscle artifact segments with a sampling rate of 250 Hz and a length of 10 s. Each individual segment is denoted by **x***_EMG_*.

**Figure 1.**
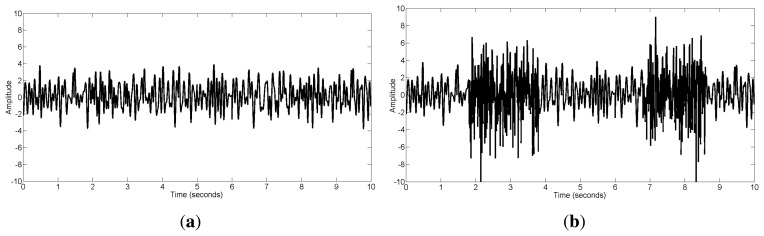
(**a**) The original EEG data; (**b**) The contaminated EEG data by muscle activity.

The EMG activity was superimposed on the EEG signal as follows:

(16) $$\text{x}={\text{x}}_{EEG}+\varepsilon {\text{x}}_{EMG}$$

where *ε* represents the contribution of muscle activity. Figure 1 shows the original EEG signal **x***_EEG_* and the EEG containing muscular artifacts **x** (*ε* = 1.5). The signal-to-noise ratio (SNR) can then be adjusted by changing the parameter *ε*:

(17) $$\text{SNR}=\frac{\text{RMS}({\text{x}}_{EEG})}{\text{RMS}(\varepsilon {\text{x}}_{EMG})}$$

where the root mean squared (RMS) value is defined as:

(18) $$\text{RMS}(\text{x})=\sqrt{\frac{1}{T}\text{x}{\text{x}}^{T}}$$

To be consistent with previous EEG denoising studies [11,12], the SNR values spread from 0.25 to 3, and each SNR value corresponds to one *ε* value. The relative root-mean-squared error (RRMSE) is used as an evaluation measure of the effects of muscular artifact cancellation, which is defined as follows:

(19) $$\text{RRMSE}=\frac{\text{RMS}({\text{x}}_{EEG}-\hat{\text{x}})}{\text{RMS}({\text{x}}_{EEG})}$$

where **x̂** is the estimated EEG signal after muscular artifact cancellation. To further measure the capability of the proposed method for preserving the original EEG signal, the correlation coefficient between the two waveforms **x***_EEG_* and **x̂** is also calculated. Hence, in this work, RRMSE and correlation coefficient (CC) serve as the main criteria for measuring the performance of muscular artifact cancellation.

#### 2.2.2. Real Data

**Figure 2.**
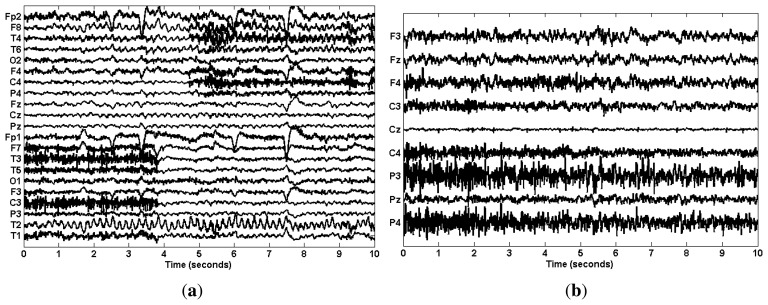
The original 10-s scalp EEG recordings for (**a**) ictal; (**b**) cycling.

For the real data case study, we used two EEG datasets. The first one was the public ictal (epilepsy) EEG data from the BioSource database established by Sabine Van Huffel (http://www.esat.kuleuven.be/stadius/members/biomed/biosource.htm). Ictal EEG signals are often severely contaminated with muscular artifacts, which make the determination and localization of the ictal onset complicated. Figure 2a shows the 10-s scalp EEG recordings with 21 channels obtained using a long-term epilepsy monitoring unit. This recording contains the ictal activity from a patient with mesial temporal lobe epilepsy. The sampling frequency was 250 Hz. The seizure activity was contaminated with muscular artifacts and eye blinks. Muscular artifacts can be observed between 0–3.9 s on channels F7, T3, T5, C3, T1 and between 5–10 s on channels F8, T4, F4, C4, P4.

The second dataset was collected from eight health subjects while stably cycling on an exercise bicycle. The EEG data were collected using an EEG cap (Quick-Cap, Compumedics, El Paso, TX, USA) with nine electrodes F3, Fz, F4, C3, Cz, C4, P3, Pz, P4 based on the International 10–20 system and using SynAmps2 amplifiers (NeuroScan, Compumedics, El Paso, TX, USA). The sampling rate was 1000 Hz. Data were later digitally band-pass filtered between 1∼70 Hz. The University of British Columbia Ethics Board approved the study. EEG recordings during cycling were easily contaminated with muscle activity, and subsequent EEG signal processing, such as brain network study, may be complicated by the resulting EMG signals. As shown in Figure 2b, all channels of the 10-s scalp EEG were contaminated with muscle activity.

Although in both cases the single-channel technique is unnecessary, we can still apply the proposed EEMD-JBSS method to each channel individually and demonstrate its effectiveness for removing muscular artifacts from different places in the brain.

## 3. Results and Discussion

### 3.1. The Synthetic Data Study

#### 3.1.1. The Real Muscle Activity Case

**Figure 3.**
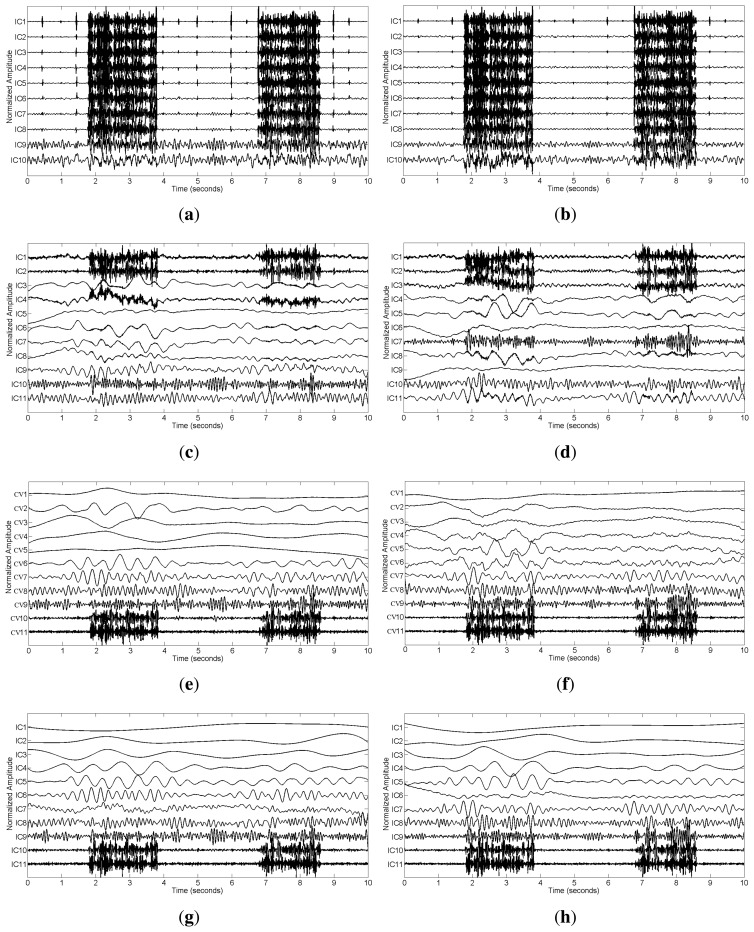
The decomposition components of the synthetic data **x** obtained using single-channel ICA (SCICA), EEMD-ICA, EEMD-canonical correlation analysis (CCA) and EEMD-independent vector analysis (IVA) at two different SNR values: (**a**) SCICA at *ε* = 2 (SNR = 0.7611); (**b**) SCICA at *ε* = 5 (SNR = 0.3044); (**c**) EEMD-ICA at *ε* = 2; (**d**) EEMD-ICA at *ε* = 5; (**e**) EEMD-CCA at *ε* = 2; (**f**) EEMD-CCA at *ε* = 5; (**g**) EEMD-IVA at *ε* = 2; (**h**) EEMD-IVA at *ε* = 5.

We applied SCICA, EEMD-ICA and the two proposed methods to the synthetic single-channel data **x**. For a complete comparison, we should compare the performance of these methods at different SNR values in terms of RRMSE and CC. However, we found that SCICA and EEMD-ICA were unable to effectively separate muscular artifacts from brain activity, e.g., at SNR = 0.76 and SNR = 0.30, as shown in Figure 3a–d. These figures present the decomposition results of SCICA and EEMD-ICA at the two different SNR values. It can be clearly seen that most components contain both muscle activity and brain activity, e.g., IC7 in Figure 3a, IC10 in Figure 3b, IC4 in Figure 3c and IC3 in Figure 3d. One possible reason is due to the fact that muscular artifacts involve the movement of a group of muscles, which do not have a stereotyped topography [12]. Thus, the two ICA-based methods do not function correctly here, as too much brain activity has to be sacrificed to be able to sufficiently remove the muscular artifacts. Moreover, it is well-known that ICA has the permutation problem and cannot return a unique result, which will increase the difficulty in the selection of artifact components during data reconstruction. Therefore, SCICA and EEMD-ICA are unsuitable for muscular artifact cancellation in the single-channel EEG case. This will be further demonstrated by real data in Section 3.2.

**Figure 4.**
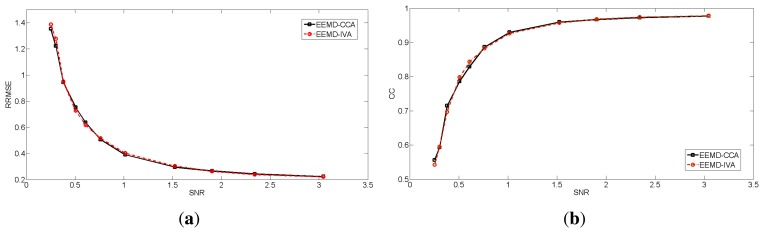
The performance measures for the two EEMD-JBSS methods at different SNR values for the real muscle activity case: (**a**) RRMSE; (**b**) CC.

In contrast, EEMD-CCA and EEMD-IVA were able to effectively isolate muscle activity into the bottom components due to their low autocorrelations, as shown in Figure 3e–h. This shows the advantage of JBSS over ICA for solving the permutation problem and facilitates automatic artifact cancellation, such as setting a threshold for autocorrelation. To compare the two EEMD-JBSS methods, we examined their performance at different SNR values in terms of RRMSE and CC, as shown in Figure 4. It can be seen that EEMD-CCA and EEMD-IVA had similar performance irrespective of the used measures. Yet, EEMD-CCA slightly outperformed EEMD-IVA when the SNR values were very low. The possible reason is that IVA assumes the underlying sources follow a Gaussian distribution, which may not be satisfied in practice. Moreover, EEMD-CCA only employs SOS and, thus, has higher computational efficiency than EEMD-IVA, which utilizes HOS. Therefore, for practical reasons, we conclude that EEMD-CCA outperformed all other methods.

**Figure 5.**
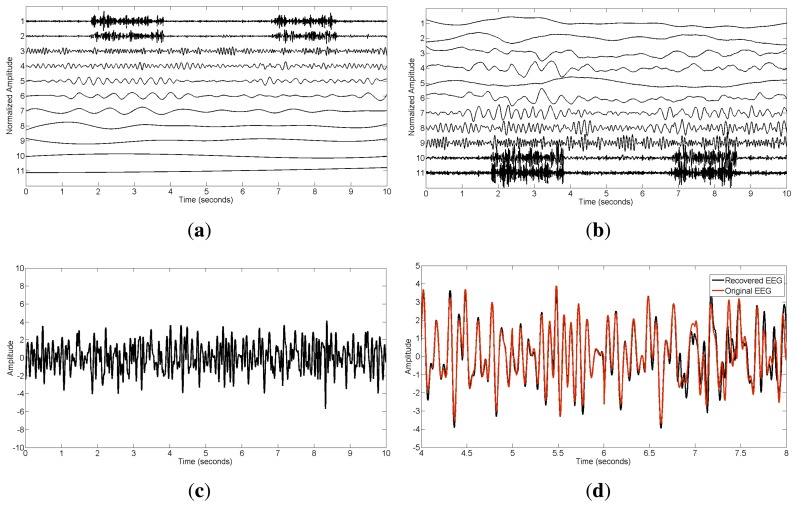
The step-wise results of the EEMD-CCA method: (**a**) the intrinsic mode function (IMF) components after applying EEMD to the single-channel EEG **x** (*ε* = 1.5); (**b**) the canonical variates after using CCA; (**c**) the reconstructed EEG signal **x̂** after muscular artifact cancellation; (**d**) the amplified version of **x̂** compared with the original EEG **x***_EEG_*.

To see more details about the EEMD-CCA method, we also present the step-wise results in Figure 5. The IMF components extracted by EEMD are shown in Figure 5a, where those with small indexes correspond to components of high frequencies and *vice versa*. After applying CCA, the uncorrelated sources were ordered in terms of their autocorrelations, as displayed in Figure 5b. Muscle activity was present in the bottom two components with lowest autocorrelations in the CCA decomposition. Excluding the muscular artifact components in the reconstruction led to the cleaned EEG shown in Figure 5c. To further illustrate the performance, an amplified version, including both recovered and original EEG signals, is presented in Figure 5d. From this figure, we can see that the proposed method highly preserved the original brain activity.

#### 3.1.2. The Simulated Muscle Activity Case

**Figure 6.**
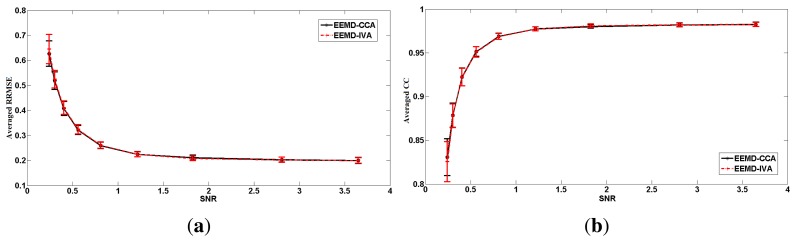
The performance measures for the two EEMD-JBSS methods at different SNR values for the simulated muscle activity case: (**a**) relative root-mean-squared error (RRMSE); (**b**) CC.

To avoid the possibly subjective comparison between EEMD-CCA and EEMD-IVA, we simulated 100 independent segments that only contained transient muscle artifacts. Each segment was 10 s long and had a 2-s transient muscle artifact. Each individual segment **x***_EMG_* was superimposed on the simulated EEG **x***_EEG_* at different SNR values. Then, we evaluated the performance of the two proposed methods on the 100 segments. We obtained 100 RRMSE values and 100 CC values for each method at each SNR value. The means and standard deviations are shown in Figure 6. Through this extensive numerical simulation, we can see that EEMD-CCA and EEMD-IVA still had almost the same performance at most SNR values. However, EEMD-CCA slightly outperformed EEMD-IVA at the lowest SNR value. The reasons are discussed in Section 3.1.1.

### 3.2. The Real Data Study

**Figure 7.**
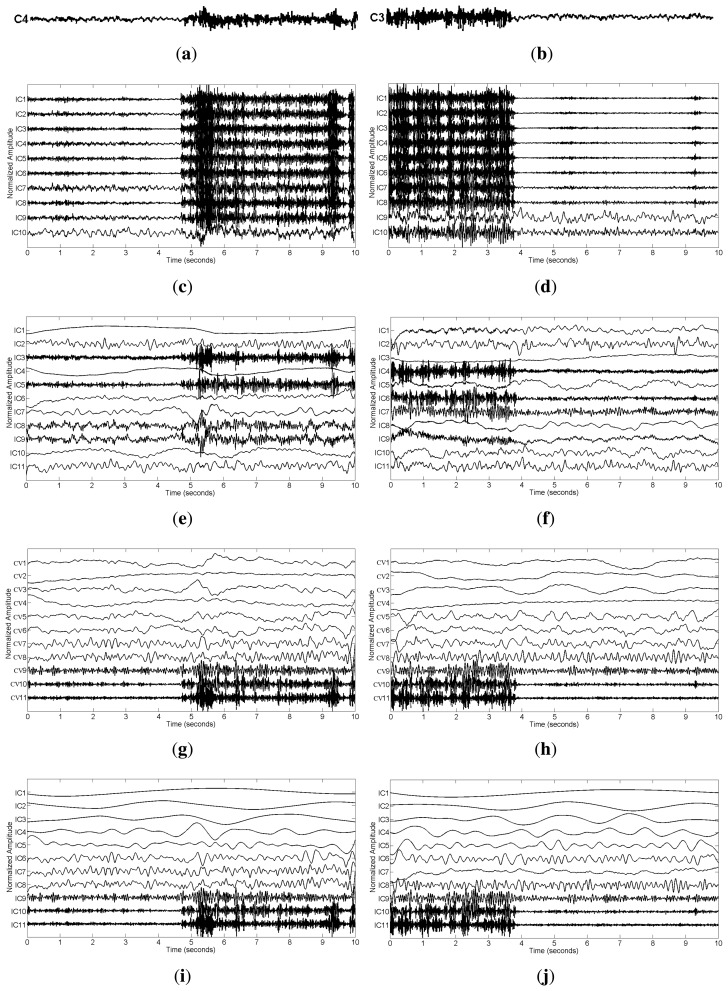
The decomposition components of EEG signals in (**a**) C4 and (**b**) C3 obtained using the four methods: SCICA for (**c**) C4 and (**d**) C3; EEMD-ICA for (**e**) C4 and (**f**) C3; EEMD-CCA for (**g**) C4 and (**h**) C3; EEMD-IVA for (**i**) C4 and (**j**) C3.

For the real data case, we first examined the ictal EEG data. The effect of muscular artifact cancellation highly depends on whether the methods can isolate muscle activity from brain activity. Hence, in Figure 7, we first present the decomposition results by applying these methods to the two channels, C4 and C3 (Figure 2a), which were severely contaminated with muscle activity.

From Figure 7c–f, we can see that SCICA and EEMD-ICA were unable to sufficiently extract the components for muscle activity; thus, it was impossible for them to remove muscular artifacts successfully. Moreover, if we attempt to remove them manually, we have to identify the components corresponding to muscle activity, which is quite time consuming and unsuitable for practical usage. However, by using EEMD-JBSS, we found that it was fairly easy to distinguish the muscular artifact components from the ones related to brain activity. As shown in Figure 7g–j, muscle activity is easily observed in the bottom two components with indexes 10–11 in the JBSS decomposition. By setting a proper threshold value for the autocorrelation, we can remove muscle activity automatically. Analogous to the simulation case, EEMD-CCA and EEMD-IVA obtained similar decomposition results.

We also tested the computational time cost of EEMD-CCA and EEMD-IVA over each of the 21 channels separately. The mean time for EEMD-CCA over each channel was 2.606 s with a standard deviation 0.0918, while the mean for EEMD-IVA was 2.756 s with a standard deviation 0.3253. The implementation was done in MATLAB (MathWorks Inc., Novi, MI, USA) and run under Microsoft Windows 8 × 64 OS on a computer with Dual Intel(R) Core(TM) i-3427U 1.80 GHz CPU and 8.00 GB RAM. The time cost is well acceptable for removing artifacts from 10-s EEG data, especially for ambulatory systems for which obtaining clean information and direct feedback in a fast fashion are essential. Considering such practical issues, EEMD-CCA becomes the best choice for this single-channel problem.

**Figure 8.**
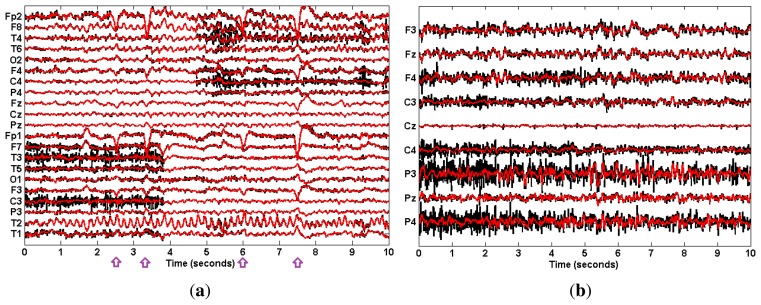
The reconstructed EEG signals after muscular artifact cancellation (red) compared with the original EEG recordings (black): (**a**) ictal; (**b**) cycling. The purple arrows indicate EOG events.

Finally, we applied the proposed EEMD-CCA method to each individual channel of the EEG recordings, as shown in Figure 2. When processing the EEG recordings of each single channel, muscle activity was present in the last two components in the ictal EEG and in the last four in the cycling EEG data. Excluding those components in the reconstruction of the EEG resulted in the cleaned EEG (red) shown in Figure 8. It can be seen that muscular artifacts were sufficiently removed, in contrast to the original EEG (black). In particular, for the ictal EEG, the ictal activity in each of the T2, F8, T4 and T6 electrodes was perfectly preserved. The ictal activity in F8 and T4, which originally was blurred by muscular artifacts, became visible by using the proposed EEMD-CCA method. It should also be noted that there existed some obvious EOG artifacts (marked) in ictal EEG, while their cancellation was beyond the scope of this paper. However, these EOG artifacts help demonstrate the superior performance of our proposed method due to the fact that they were preserved with little distortion.

**Figure 9.**
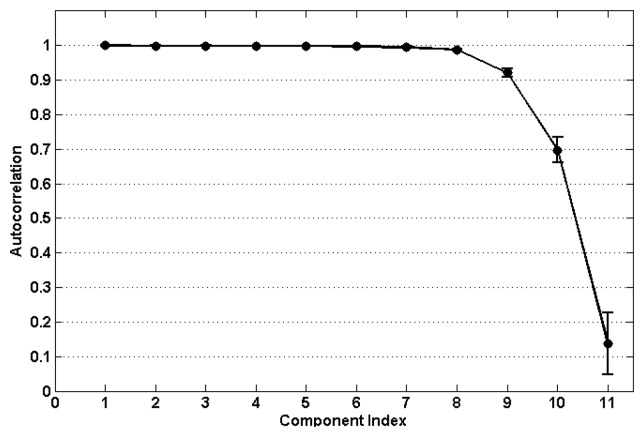
The averaged autocorrelation of the eleven EEMD-CCA components over all 21 ictal EEG channels.

To provide some practical guidance for the selection of the autocorrelation threshold value, we calculated the autocorrelation values for the eleven decomposition components of each individual ictal EEG channel. Figure 9 presents the mean and standard deviation values averaged across the 21 ictal EEG channels. We suggest that the threshold should be set to no less than 0.9. The components with the value below 0.9 are deemed to be muscle artifacts.

## 4. Conclusions

As the popularity of using ambulatory devices in healthcare systems increases, more applications that rely on EEG signals are being developed. For practical reasons, these applications use only one EEG channel. For such ambulatory applications, muscle artifact removal from the EEG recordings becomes important. Although there exist two IC-based methods for performing source separation of a single-channel signal, they are found unsuitable for removing the artifacts arising from muscle activity. In this paper, we propose two effective methods for canceling muscular artifacts in single-channel EEG recordings. Each method utilizes the known EEMD and JBSS techniques, and they are denoted as EEMD-CCA and EEMD-IVA. Their implementation has two steps. In the first step, EEMD is used to decompose the single-channel EEG into multichannel datasets. In the second step, CCA or IVA is applied to separate the muscle artifacts from the multidimensional datasets obtained in the first step. The main difference between CCA and IVA is that CCA utilizes second order statistics, while IVA employs higher-order statistics and assumes a specific probability distribution. We examined the performance of the two proposed methods using synthetic data, as well as real-life data. We observed that both proposed methods were able to remove muscle activity while also preserving the brain activity very well. The performance of EEMD-CCA was, however, slightly better than that of EEMD-IVA, and its computational efficiency was significantly better. Therefore, EEMD-CCA is recommended in this work. It is worth noting that besides being effective in removing muscle activity in the single-channel EEG case, EEMD-CCA is also applicable in the multichannel case when few channels (e.g., two or three) are used.
